# Microleakage of Lithium Disilicate Veneers Bonded to Different Substrates with Light-cure and Dual-cure Resin Cements

**DOI:** 10.4317/jced.61279

**Published:** 2024-04-01

**Authors:** Bahil-Imad Albaheli, Mohamed-Elshirbeny Elawsya, Ashraf-Ibrahim Ali

**Affiliations:** 1 Postgraduate MSc student, Department of Conservative Dentistry, Faculty of Dentistry, Mansoura University, Egypt; 2 Lecturer, Department of Conservative Dentistry, Faculty of Dentistry, Mansoura University, Egypt; 3 Associate Professor, Department of Conservative Dentistry, Faculty of Dentistry, Mansoura University, Egypt

## Abstract

**Background:**

This study aimed to evaluate the microleakage of lithium disilicate veneers with finish lines placed cervically in different substrates (enamel, dentin, and resin composite) and bonded with light-cure (LC) and amine-free dual-cure (DC) resin cements.

**Material and Methods:**

Forty-eight human maxillary central incisors were randomly assigned into three groups according to finish line substrate (n=16/group). Each group was subdivided randomly into two subgroups (n=8/subgroup) according to resin cement type: LC resin cement (Variolink Esthetic LC, Ivoclar Vivadent) and DC resin cement (Variolink Esthetic DC, Ivoclar Vivadent). All the specimens received lithium disilicate veneers (IPS e.max Press, Ivoclar Vivadent). After 5000 cycles of thermocycling, the microleakage was measured using the dye penetrating technique. Data were analyzed statistically using Scheirer Ray Hare test, Kruskal-Wallis H-test, and Mann-Whitney U-test. The level of significance was set at *p* ≤ .05.

**Results:**

There was a statistically significant difference between different substrates in microleakage (*p*=.001), but there was no statistically significant difference between resin cements (*p*=.907), and there was no interaction between substrates and resin cements (p=.983). Microleakage was lesser when the finish line was placed at enamel and resin composite than at dentin. Similar leakage scores were observed with LC and DC resin cements.

**Conclusions:**

The finish line of ceramic veneer is suggested to be placed in enamel or good-quality resin composite restoration. Regarding microleakage and durability, LC and amine-free DC resin cements are suggested for ceramic veneer cementation.

** Key words:**Different substrates, Dual-cure resin cement, Light-cure resin cement, Lithium disilicate veneers, Microleakage.

## Introduction

All-ceramic restorations have gained popularity in the last two decades due to their ability to restore and mimic the natural teeth appearance and provide good esthetic results with high survival rates, making it indicated for veneers, inlays, onlays, and full-coverage crowns (1,2). Ceramic veneers are type of dental prostheses utilized to restore the facial and part of the proximal surfaces of anterior teeth. These thin veneers are bonded and made from glazed ceramic materials, representing a durable anterior esthetic restoration (3). Indeed, they are the first solution for teeth suffering from severe discoloration that doesn’t resolve by bleaching, enamel defects, diastemas and black triangles, mal-positioned or fractured teeth, and aging (4). Various materials, preparation designs, fabrication methods, and bonding techniques are available for ceramic veneers, and the clinician should seek the best treatment framework according to the clinical situation to provide restoration with better longevity and survival rates. Ceramic veneers of 0.3-0.5 mm thickness decrease tooth reduction in a minimally invasive procedure (5,6).

The success of any ceramic restoration is dependent on many factors, including its optical, mechanical, and bonding properties as well as marginal fit and microleakage (7). Microleakage is the entry of fluids, microbes, ions, or chemicals through the interface connecting the tooth and the restoration that could lead to marginal discoloration, hypersensitivity, or even recurrent caries and has been used to assess the clinical success of cemented restorations for many years (8,9).

LC resin cements are the first choice for thin ceramic veneer cementation due to their durable working time and color stability. Traditional DC resin cements have shown superior mechanical properties and a high degree of conversion. However, they are unfavorable for ceramic veneer cementation because of their limited working time and lower color stability (due to aromatic tertiary amines in their formulation as coinitiator) that produce a yellowing effect on the resin material in the long term (10). New DC resin cements that don’t depend on tertiary amines in their polymerization reaction promise to be indicated for cementing ceramic veneers that give better color stability, provided with try-in pastes for better esthetic outcomes as in LC resin cements, and also have a greater bond strength than LC resin cements (11-13).

As ceramic veneer restorations do not rely on their macro-mechanical retention, the bond of ceramic to tooth structure has to be optimal to resist shear forces during oral function. A reliable bond strength can be obtained between the veneers and enamel because of the elevated mineral concentration and reduced water content. However, sometimes the excessive offering of dental treatment in the trend for using ceramic veneers for so-called “Instant Orthodontics” results in preparing the teeth deeper than guidelines, results in significant destruction of teeth and the exposure of dentin as a substrate for adhesion, a condition characterized by more significant variability because of increased moisture and greater technique sensitivity. In certain clinical scenarios, applying the margins on direct composite restorative materials within the aesthetic zone may be necessary. This is because the total removal of preexisting composite restorations could potentially result in the enlargement of cavities, leading to additional loss of tooth structure (4,14).

Several studies evaluated the microleakage of ceramic veneers with the finish line placed in enamel and dentin (4,15,16). Still, only a few conflicting studies evaluated microleakage with the finish line placed in resin composite (14,17). Moreover, a study in the literature confirms that LC and DC resin cements show a similar leakage pattern in ceramic veneers (18). On the contrary, another study states that DC resin cements reported less microleakage with veneers (19). Considering these conflicts, the comparative evaluation of microleakage in ceramic veneers cemented to different substrates with different resin cements still needs further investigation, especially with the development of new, improved resin cements with new formulations and polymerization techniques.

Hence, this study was planned to evaluate the microleakage of ceramic veneers with finish lines placed cervically in enamel, dentin, and resin composite restoration and cemented with LC and amine-free DC resin cements. The first null hypothesis tested was that the bonding cervical finish line substrate would not affect the microleakage of ceramic veneers. The second null hypothesis tested was that the resin cement type would not affect the microleakage of ceramic veneers.

## Material and Methods

-Materials

All materials used in this study and their descriptions are presented in (Table 1).

**Table 1:**
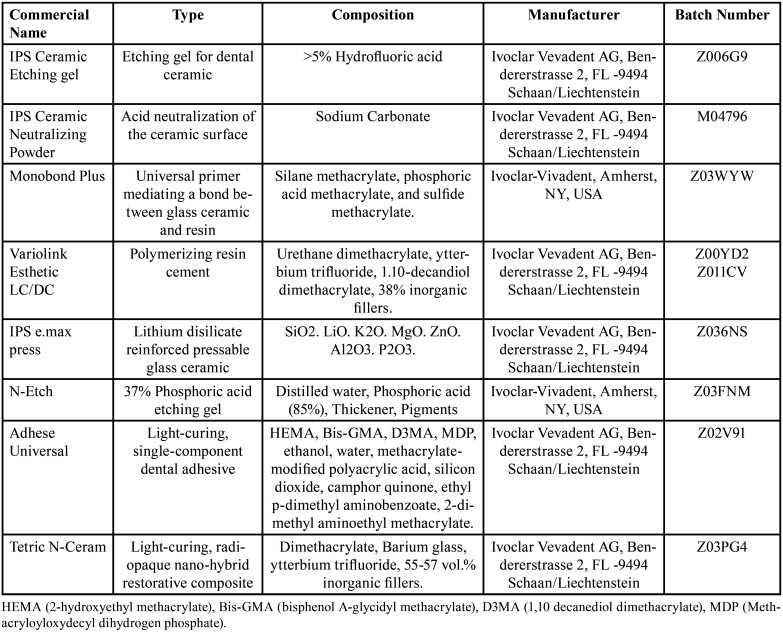
Materials used in the study.

-Sample size calculation

Assuming a power of 80% and a significance level of 0.05, the sample size was calculated using G*Power 3.1 (Heinrich Heine University, Düsseldorf, Germany) based on the results of a similar study design conducted by Alnakib Y *et al*. (20). According to this calculation, the minimum sample size required per subgroup is 8.

-Study design and specimen preparation

The Dental Research Ethics Committee (Faculty of Dentistry, Mansoura University) approved this study under protocol number (A06061222). Forty-eight sound human maxillary central incisors, freshly extracted due to periodontal problems were collected. The root apices were blocked by glass ionomer cement (MICRON Luting; Prevest DenPro Limited, Bari Brahmana, India). The specimens were randomly assigned to three main groups according to cervical finish line substrate (n=16/group): Group A: finish line placed in enamel; Group B: finish line placed in dentin; Group C: finish line placed in Class V resin composite restoration. Each group was subdivided randomly into two subgroups (n=8/subgroup) according to resin cement type (LC and DC resin cements).

Subsequently, the specimens were mounted vertically in auto-polymerizing resin blocks (Acrostone, Cairo, Egypt). In the subsequent study procedure, the specimens were stored in distilled water at 37±1 °C in an incubator (Model: BT1020, BTC, Cairo, Egypt) and refreshed regularly with new water every five days during the study to prevent dehydration. The same operator did all the preparations using a high-speed handpiece with diamond burs (Prep-Set, Intensiv, Viaganello-Lugano, Switzerland) under constant copious water coolant irrigation. Diamond burs were changed regularly after each five preparations.

-Class V cavity preparation and restoration 

Regarding group C, specimens received standardized conventional Class V cavities labially (3.5 mm width, 3 mm height, and 2 mm depth). The coronal margins were located 2 mm in enamel, and the cervical margins were located 1 mm in cementum. All cavities were restored following the manufacturer’s instructions. The enamel parts of the cavity margins were etched with 37% phosphoric acid etching gel (N-Etch; Ivoclar-Vivadent, Amherst, NY, USA) for 30 seconds, followed by a rinsing period of 15 seconds and subsequent drying. Single-component dental adhesive (Adhese Universal; Ivoclar Vivadent, Shaan, Liechtenstein) was applied with a disposable brush and well-dried and light-cured using poly-wave light-emitting diode (LED) light curing unit (LCU) (Bluephase G2; Ivoclar Vivadent). Subsequently, restored using a LC nano-hybrid composite (Tetric N-Ceram; Ivoclar Vevadent AG, Schaan, Liechtenstein) in two horizontal layers and light-cured. Another curing circle was done after applying a glycerin gel (Liquid Strip, Ivoclar Vivadent AG, Schaan, Liechtenstein) as an oxygen-inhibition layer. The resin composite restorations were finished and polished using Dicomp plus TWIST finishing and polishing kit (EVE Emst Vetter GmbH, Neureutstr, Germany).

-Veneer preparation 

Before preparation, labial and lingual silicon indexes were constructed with putty condensation silicone (Virtual, Ivoclar Vevadent AG, Schaan, Liechtenstein) to standardize the preparation and to provide a visual reference at least three times during preparation. For incisal reduction, a butt-joint preparation design was performed with a 2 mm reduction of the incisal edge by creating grooves in the incisal edge with a 2 mm special depth marker bur (#707C), then 1 mm length wheel bur (#110C) was used to complete the incisal reduction without palatal chamfer.

For axial reduction, a pilot bur (#S4) was used for depth specification of 0.4 mm labial reduction to ensure a uniform reduction over the labial surface. The preparation outlines were painted on the tooth with a waterproof color marker to create a visual reference for the preparation area. The labial tooth reduction was done with a round-end tapered diamond bur (#233C). The finish line was placed 1 mm coronal to CEJ in groups A and C, and 1 mm cervical to CEJ in group B. It was created with 0.5 mm chamfer and continued interproximally without breaking the mesial and distal contacts. All the line angles were smoothly curved, and all the margins were finished using cylindrical finishing burs (#4307N) and (#4192).

-Impression taking and veneer fabrication

All specimens’ impressions were fulfilled under extraoral conditions using a HERON intraoral scanner (3 DISC; 3D Imaging and Simulations Corp. Americas, United States). To produce a digital design, these digital Polygon files (PLY) data were used in CAD software (exocad Dental CAD; 3.1 Rijeka, Darmstadt, Germany).

After acceptance of the design, standard tessellation language (STL) files were transferred from the CAD software to the CAM software (ceramill motion; Amann Girrbach AG, Koblach, Austria), and from that design, the CAD wax patterns were milled with a 5-axis milling machine (Ceramill mikro; Amann Girrbach AG, Koblach, Austria) using milling wax disc Aidite WAX 98*18 Blue (Aidite, Economic and Technological Developmental Zone, Qinhuangdao City, Hebei Province, China). Then all the wax patterns were sprued and invested. Lithium disilicate reinforced pressable glass ceramic ingots (IPS e.max Press) were pressed in a press furnace (Programat EP5010; Ivoclar Vevadent AG, Schaan, Liechtenstein). Finally, the veneers were glazed and cleaned ultrasonically for 10 minutes before cementation.

-Cementation and finishing

The fitting surface of the veneer was treated according to manufacturer instructions. Started with etching for 20 seconds using >5% hydrofluoric acid (IPS Ceramic Etching Gel; Ivoclar Vevadent AG, Schaan, Liechtenstein) followed by rinsing, then sodium carbonate (IPS Ceramic Neutralizing powder; Ivoclar Vevadent AG, Schaan, Liechtenstein) was applied for five minutes to neutralize the diluted solution of etching gel and water. An ultrasonic path with 95% alcohol was used for four minutes so all the residual acid and dissolved debris were eliminated, followed by rinsing and dryness. Subsequently, a thin coat of universal primer containing silane mediating a mechanical and chemical bond between glass ceramic and resin (Monobond Plus; Ivoclar-Vivadent, Amherst, NY, USA) was applied using a disposable brush on the etched surface and allowed to react for 60 seconds. Any remaining excess was dispersed with a strong stream of air.

The prepared surface of the specimen was treated following the manufacturer’s recommendations. Group C started with sandblasting the resin composite restoration surface with 50 µm aluminum oxide particles for 10 seconds and 10 mm away from the surface at 60 psi, using a sandblasting unit (Microetcher, Danville Materials, San Ramon, CA, USA). After that, the conditioning of the whole surface of the preparation in all groups was conventionally carried on according to the selective enamel etching procedure with Adhese Universal without light curing. Resin cement (Variolink Esthetic LC/DC) was applied to the veneer fitting surface and cemented on the prepared tooth surface. Cementation standardization in this study is performed by a single operator who proceed the same cementation steps for all specimens according to manufacturer instructions. Veneers were seated with gentle and gradual finger pressure on the mid-third of the veneer along the insertion axis until complete seating of the veneer.

Excess cement was light cured by Bluephase G2 for only two seconds using a circular technique with start and end points in the incisal area following the cement line by moving the polymerization light in a circle in a clockwise direction at a distance of 10 mm. This was followed by the subsequent removal of excess cement by running a probe along the entire cement line with the tip of the probe guided parallel to the margins to avoid extraction of resin cement from the margins, followed by elimination of the last excess of resin cement with a clean disposable brush moistened with Adhese Universal to avoid marginal gap and provide polished margins. Later, light curing started for 60 seconds of irradiation for each surface, in 20-second intervals, and then a layer of Liquid Strip was applied for the margins, followed by additional curing. Subsequently, the Liquid Strip was easily removed, and any excess adhesive and cement were removed with a hand instrument using a surgical blade scalpel (#12).

-Thermocycling

All the specimens were subjected to thermocycling using a thermal cycling device (The mechatronic thermocycler, Germany) for a total number of 5,000 cycles between 5 ± 2°C and 55 ± 2°C to mimic the thermal variations that occur within the oral cavity with a rest period of 20 seconds for each bath and 10 seconds interval between baths at room air temperature.

-Microleakage test

**Figure 1:**
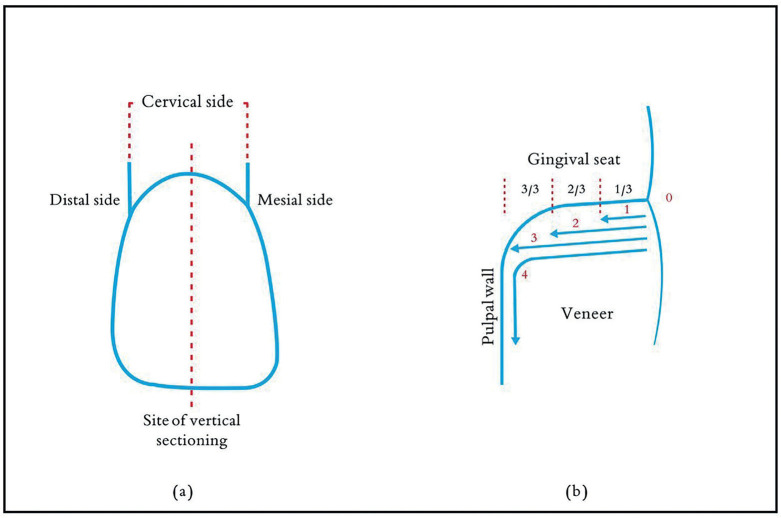
(a) Vertical specimen sectioning, (b) Microleakage scores for dye penetration.

All specimens were coated with double layers of nail varnish, except a 1 mm border around the cervical margin of the veneer, to allow dye contact with this margin. Subsequently, the specimens were placed in a 2% methylene blue penetrating solution for 24 hours, then withdrawn from the dye and washed with running water; afterward, specimens were dried for another 24 hours (21). Later, the specimens were sectioned in a bucco-lingual direction using a low-speed diamond saw (Isomet 1000, Buehler, Lake Bluff, IL, USA) with water-cooling. The cuts were made parallel to the long axis of the tooth and were positioned at the midpoint of the interproximal dimension of the cervical margin of the veneers (Fig. 1a).

**Figure 2:**
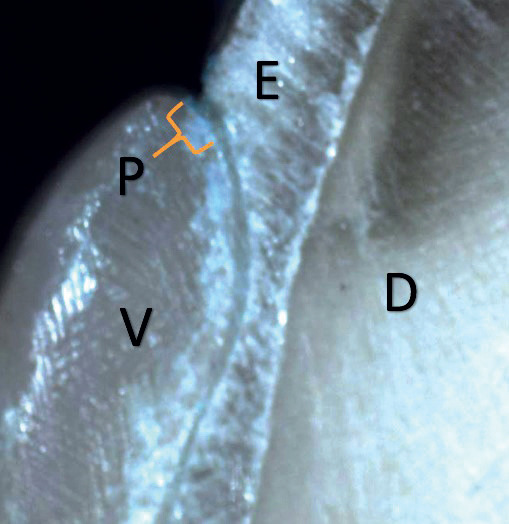
Score 1 for dye penetration (group A with LC resin cement). E: enamel, D: dentin, V: veneer, P: dye penetration.

A single trained clinician evaluated all the specimens, and the presence of microleakage was confirmed by the visualization of a blue dye extent into the resin cement/tooth substrate interface cervically by a stereomicroscope (Nikon Stereo Microscopes-SMZ1500) under 35X magnification in a blind manner based on a scoring system similar to that used by Jia S *et al*. (21), (Fig. 1b). Value and its inference used in the present study were as follows: 0: no penetration of the dye, 1: penetration of the dye up to the first third of the gingival seat, 2: penetration of the dye up to the second third of the gingival seat, 3: penetration of the dye into the entire gingival seat, 4: penetration of the dye into the whole of the gingival seat and extend to the pulpal wall. The worst score of the two sections of each specimen was used (Fig. 2). The evaluation process was done again after a 24-hour interval by the same clinician, who kept unaware of the prior scores. The scores were afterwards calculated, and the ultimate data was gathered from each scoring of the specimens, utilizing the lowest value for each scoring.

-Statistical analysis

Data were entered and analyzed using IBM-SPSS software (IBM Corp. Released 2020. IBM SPSS Statistics for Windows, Version 27.0. Armonk, NY: IBM Corp). After exploring the data distribution (Shapiro-Wilk test), data showed a non-parametric distribution. Qualitative data were expressed as N (%). Ordinal data were described as median and range (minimum–maximum). The Mann-Whitney U-test was used to compare ordinal non-parametric data between the two groups. The Kruskal-Wallis H-test was used to compare ordinal non-parametric data between more than two groups. The Scheirer Ray Hare test is the two-factor version of the Kruskal-Wallis H-test. The level of significance was set at *p* ≤ .05.

## Results

**Figure 3:**
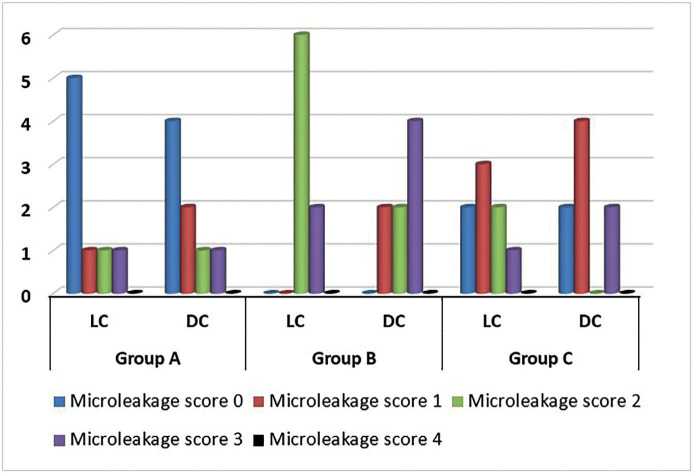
Bar chart for the frequencies of microleakage scores in all groups.

The Scheirer Ray Hare test used the Real Statistics Data Analysis Tool (The Real Statistics Resource Pack in Excel). The rows factor represented finish line substrates (three levels: enamel, dentin, and resin composite), while the columns factor represented the resin cement types (LC and DC resin cements) (Table 2). Regarding microleakage, there was a statistically significant difference between the different substrates (*p*=.001), but there was no statistically significant difference between the two resin cements (*p*=.907), and there was no interaction between different substrates and the used resin cements (*p*=.983). Frequencies of microleakage scores in each group are presented in (Fig. 3).

**Table 2:**
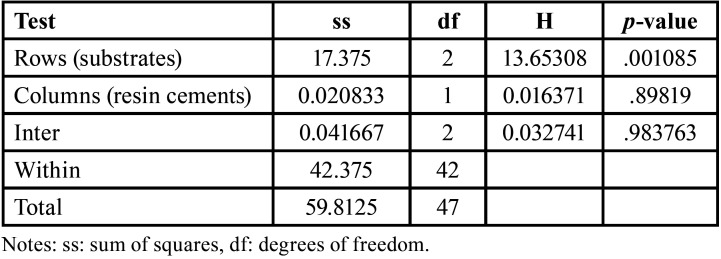
Scheirer Ray Hare test for microleakage.

Microleakage scores were higher in dentin, followed by resin composite restoration, and less in enamel. The Kruskal-Wallis H-test indicated that the differences between enamel vs dentin and dentin vs resin composite restoration were statistically significant (*p*<.05). In contrast, the difference between enamel vs resin composite restoration was not statistically significant (*p*>.05) (Table 3). The Mann-Whitney U-test indicated that there was no statistically significant difference between the two resin cements (*p*>.05) (Table 4).

**Table 3:**
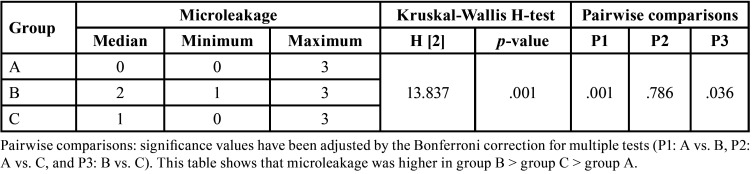
Microleakage comparisons between the three substrate groups.

**Table 4:**
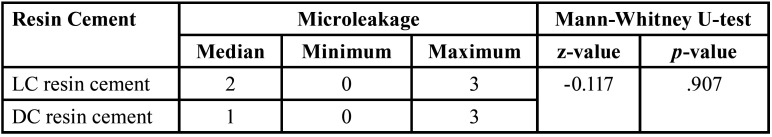
Microleakage comparisons between the two resin cements.

## Discussion

The success and longevity of ceramic veneers depend on several factors; the significant reasons for indirect restoration failure are microleakage, loss of retention due to adhesion cement shrinkage, and dissolution, which can be minimized by better marginal adaptation (8). Due to the polymerization shrinkage of resin cement and the difference in the coefficient of thermal expansion of the linked interfaces, stresses will result, which leads to competition between the adhesive forces of the two bonded interfaces. The failure occurs with the lowest adhesive forces, which are in the resin cement/ tooth substrate interface because the ceramic surface is more etchable than the tooth substrate. Microleakage was tested at the resin cement/ tooth substrate interface only and the evaluation was done at the cervical margin because it is more pronounced, which may be due to the increased occurrence of marginal gaps cervically by heavy occlusal stress during normal function and parafunction, which causes the tooth to flex (22).

Regardless of the selected resin cement, the cervical finish line substrate significantly affected ceramic veneers’ microleakage. When the three analyzed finish line substrates were compared, there was no significant difference between groups A and C. However, group B exhibited significantly higher microleakage scores than other groups, and these results agree with various microleakage studies (4,15,16). Maybe because of wider biological variations and organic/inorganic composition of dentin compared to enamel makes it more challenging to create a strong adhesive to resist stresses of thermocycling and polymerization shrinkage of resin cement (17).

Group C presented no significantly higher microleakage in resin composite/ resin cement interface than group A. These results agree with Cavalcanti A *et al*. (23). who showed little or no dye penetration at the repair interface between two resin composites and agree with the results of a clinical study by Gresnigt M *et al*. (14). who showed that pre-existing restorations do not affect the survival rate of ceramic veneers. The results disagree with Metz *et al*. (17). who found that finish line in resin composite had significantly higher microleakage at the resin composite/ resin cement interface than the finish line in enamel. This study may have provided better results in resin composite substrate due to the roughening of the restoration by sandblasting and using nanohybrid resin composite with the corresponding universal adhesive system (24). Regarding the resin cement used, there was no statistically significant difference in microleakage between LC and DC resin cements. These results support the opinion of Zaimoğlu A. *et al*. (18). maybe because of similar filler loading, coefficient of thermal expansion, and polymerization shrinkage of both resin cements.

This study used the polywave LED LCU (Bluephase G2) to ensure adequate material polymerization and achieve excellent mechanical properties of the resin cement because Variolink Esthetic LC and Variolink Esthetic DC resin cements have Ivocerin as their photoinitiator, which is primarily sensitized by wavelengths range of (380-420 nm) making it essential to use polywave LCU (10,25). The preparation design used was butt-joint with an incisal reduction without palatal chamfer to provide a simplified preparation technique for better standardization and less marginal gap. Preparation was finished, and all sharp angles were removed to improve the quality of preparation and impression, which facilitated the work and led to a minimal cement gap (6). The intraoral scanner was used as an alternative to the conventional method for better standardization, simplicity, and significantly higher accuracy (26). Pressable ceramic was used for better marginal adaptation, minimal cement gap, and less microleakage than machinable ceramic veneers (27). The milling waxing technique was used to provide standardization and simplicity at the same marginal and internal fit quality as a manual waxing technique (28). Thermocycling was described in the current study to simulate the oral condition as closely as possible. For microleakage testing, methylene blue dye penetration and cross-section technique was selected in the current study because it is the most commonly used by a large number of studies, simple, cost-effective, and has a high degree of staining and a molecular weight similar to butyric acid, a microbial metabolic product with greater penetration than Indian ink (8,29).

According to the results of this study, the first null hypothesis, namely that the three substrates are equally effective against microleakage was rejected, as there was a statistically significant difference in microleakage between the three substrates. However, the second null hypothesis, namely that the two resin cements are equally effective against microleakage was accepted, as there was no statistically significant difference in microleakage between LC and DC resin cements. In this study, the role of mechanical loading was not considered, which is a limitation of this study. Thus, further studies are necessary considering the role of mechanical loading on ceramic veneers’ microleakage bonded to the three different substrates with LC and DC resin cements and to evaluate the microleakage of ceramic veneers bonded with a finish line placed in resin composite, with different surface treatments, bonding procedures, or even different resin cements.

## Conclusions

Despite the limitations of this study, it can be concluded that:

1. Microleakage was lesser when the cervical finish line of lithium disilicate veneer was placed at enamel and resin composite than at dentin.

2. Similar leakage scores were observed when LC and DC resin cements were used.
